# One molecular fingerprint to rule them all: drugs, biomolecules, and the metabolome

**DOI:** 10.1186/s13321-020-00445-4

**Published:** 2020-06-12

**Authors:** Alice Capecchi, Daniel Probst, Jean-Louis Reymond

**Affiliations:** Department of Chemistry and Biochemistry, University of Bern, Freiestrasse 3, 3012 Bern, Switzerland

**Keywords:** Molecular fingerprints, Virtual screening, Chemical space, Databases, Locality sensitive hashing

## Abstract

**Background:**

Molecular fingerprints are essential cheminformatics tools for virtual screening and mapping chemical space. Among the different types of fingerprints, substructure fingerprints perform best for small molecules such as drugs, while atom-pair fingerprints are preferable for large molecules such as peptides. However, no available fingerprint achieves good performance on both classes of molecules.

**Results:**

Here we set out to design a new fingerprint suitable for both small and large molecules by combining substructure and atom-pair concepts. Our quest resulted in a new fingerprint called MinHashed atom-pair fingerprint up to a diameter of four bonds (MAP4). In this fingerprint the circular substructures with radii of *r* = 1 and *r *= 2 bonds around each atom in an atom-pair are written as two pairs of SMILES, each pair being combined with the topological distance separating the two central atoms. These so-called atom-pair molecular shingles are hashed, and the resulting set of hashes is MinHashed to form the MAP4 fingerprint. MAP4 significantly outperforms all other fingerprints on an extended benchmark that combines the Riniker and Landrum small molecule benchmark with a peptide benchmark recovering BLAST analogs from either scrambled or point mutation analogs. MAP4 furthermore produces well-organized chemical space tree-maps (TMAPs) for databases as diverse as DrugBank, ChEMBL, SwissProt and the Human Metabolome Database (HMBD), and differentiates between all metabolites in HMBD, over 70% of which are indistinguishable from their nearest neighbor using substructure fingerprints.

**Conclusion:**

MAP4 is a new molecular fingerprint suitable for drugs, biomolecules, and the metabolome and can be adopted as a universal fingerprint to describe and search chemical space. The source code is available at https://github.com/reymond-group/map4 and interactive MAP4 similarity search tools and TMAPs for various databases are accessible at http://map-search.gdb.tools/ and http://tm.gdb.tools/map4/.
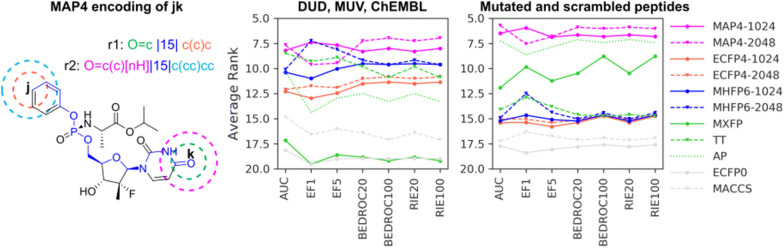

## Introduction

The diversity and size of the organic molecules of possible interest as drugs steadily increases as medicinal chemistry addresses ever more complex biological processes while also exploiting the expanding scope of synthetic organic chemistry [1–3]. Cheminformatics enables the exploitation and understanding of this diversity by describing molecules as molecular fingerprints, encoding their structural characteristics as a vector [4, 5]. These fingerprints can be used for fast similarity comparisons forming the basis for structure–activity relationship studies, virtual screening, and the construction of chemical space maps [6–9].

Most molecular fingerprints have been conceived, validated, and used in the context of small molecule drugs within the classical Lipinski limits [10], and are not well suited to describe larger molecules. For instance, the most popular molecular fingerprint is the Morgan fingerprint [11], also known as extended-connectivity fingerprint ECFP4 [12]. ECFP4 belongs to the best performing fingerprints in small molecule virtual screening [13] and target prediction benchmarks [14, 15], together with the related MinHashed fingerprint MHFP6 [16]. Both fingerprints perceive the presence of specific circular substructures around each atom in a molecule, which are predictive of the biological activities of small organic molecules. However, both have a poor perception of the global features of molecules such as size and shape. They also fail at perceiving structural differences that may be important in larger molecules, such as distinguishing between regioisomers in extended ring systems (e.g. 2,7- versus 2,8-dichlorodioxin), between linkers of different lengths, or between scrambled peptide sequences of identical composition and length.

The above limitations can be addressed by using atom-pair fingerprints [17], which encode molecular shape and are often used for scaffold-hopping [18–20]. We have shown that atom-pair fingerprints are suitable to describe large molecules by mapping the Protein DataBank [21]. We also used atom-pair fingerprints to discover and optimize novel antimicrobial peptides in virtual libraries of bicyclic peptides [22, 23] and peptide dendrimers [24, 25], to create chemical space maps [26] of molecules beyond the Lipinski limit found in the PubChem and ChEMBL databases [27], and to drive a genetic algorithm to produce analogs of peptides with diverse chain topologies [28]. Overall, atom-pair fingerprints have an excellent perception of molecular shape for both large and small molecules and overcome the above-mentioned limitations. However, they do not encode molecular structure in detail and perform poorly in small molecule benchmarking studies compared to substructure fingerprints such as ECFP4 and MHFP6.

Here we set out to investigate if the atom-pair approach could be combined with circular substructures as implemented in the above mentioned MinHashed fingerprint MHFP6 to create a new fingerprint suitable for small molecule virtual screening but also capable of describing large molecules including biopolymers such as peptides. Such a fingerprint would provide an elegant unified description of molecules across very different sizes and might also be useful to describe molecules of intermediate size such as large natural products and metabolites. Our quest uncovered a new fingerprint which we call MAP4 (MinHashed Atom-Pair fingerprint up to four bonds). MAP4 encodes atom pairs and their bond distance similarly to the AP fingerprint implemented by RDKit [29], however in MAP4 atom characteristics are replaced by the circular substructure around each atom of the pair, written in SMILES format. MAP4 uses the same MinHashing technique as MHFP6, a principle borrowed from natural language processing which enables fast similarity searches in very large databases by locality sensitive hashing (LSH). LSH is a technique that allows the creation of self-tuning indexes, which are then used to generate a forest of trees that can be traversed for an approximate but fast similarity search [30–32].

We show that MAP4 outperforms substructure fingerprints in small molecule benchmarking studies [13] and at the same time outperforms other atom-pair fingerprints in a peptide benchmark designed to evaluate performance on large molecules. Furthermore, we show with the example of various interactive tree-maps (TMAPs) [33] that MAP4 has excellent properties to map the chemical space of databases of molecules of interest across the life sciences such as bioactive molecules of various sizes (DrugBank [34], ChEMBL [35], non-Lipinski ChEMBL) [27], peptides (peptides up to 50 residues from SwissProt) [36, 37], and metabolites (Human Metabolome database) [38].

## Methods

### Fingerprint calculation

**Fig. 1:**
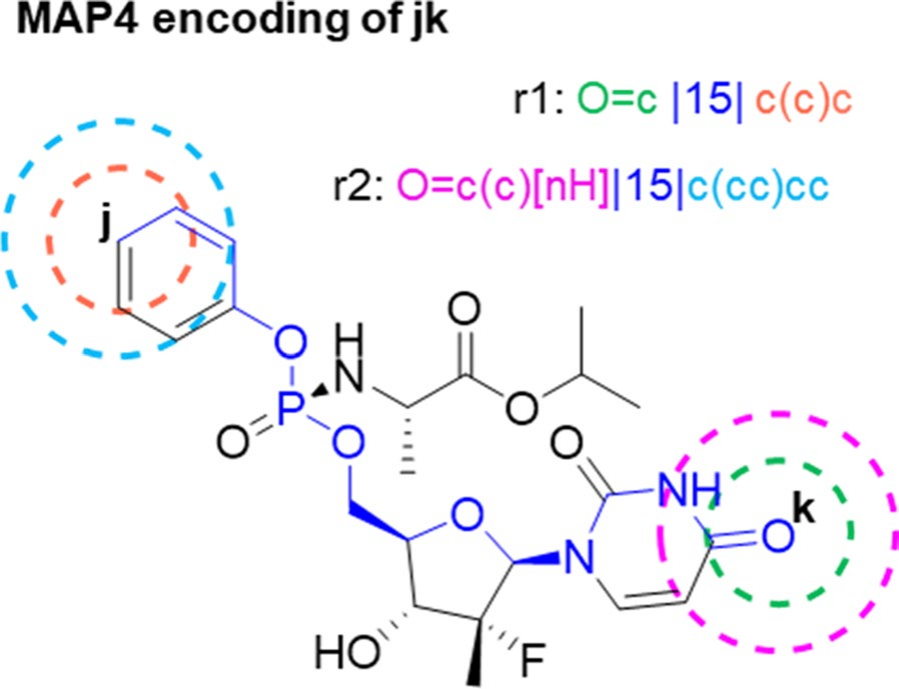
MAP4 atom pair encoding. The circular substructures around atoms *j* and *k* at radius *r* = 1 and *r* = 2 are written as SMILES placed in lexicographical order separated by the bond distance between the two atoms along the shortest path (blue). These character strings are the atom-pair molecular shingles for this atom-pair for *r* = 1 and *r* = 2

The MinHashed Atom Pair (MAP) fingerprint calculation requires a canonical and anisomeric SMILES representation of the input molecule, as well as the parameter *r*, which signifies the maximal radius of the circular substructures to be considered (default radius value *r* = 2 corresponding to a diameter *d* = 4 for MAP4). The fingerprint is calculated as follows: First, the circular substructures surrounding each non-hydrogen atom $j$ in the molecule at radii 1 to *r* are written as canonical, non-isomeric, and rooted SMILES string $CS_{r} \left( j \right)$ using RDKit [39]. Second, the minimum topological distance $TP_{j,k}$ separating each atom pair $\left( {j,k} \right)$ in the input molecule is calculated. Third, all atom-pair shingles $CS_{r} \left( j \right)\left| {TP_{j,k} } \right|CS_{r} \left( k \right)$ are written for each atom pair $\left( {j,k} \right)$ and each value of $r$, placing the two SMILES strings $CS_{r} \left( j \right)$ and $CS_{r} \left( k \right)$ in lexicographical order (Fig. 1). Fourth, the resulting set of atom-pair shingles is hashed to a set of integers $S_{i}$ using the unique mapping SHA-1 [40], and its corresponding transposed vector $S_{i}^{T}$ is finally MinHashed to form the MAP4 vector (Eq. 1). A detailed description of the MinHash method used here can be found in our recent publication on MHFP6 [16].

$$ {\text{col\_}}min \to {\text{returns}}\;{\text{the}}\;{\text{smallest}}\;{\text{number}}\;{\text{in}}\;{\text{each}}\;{\text{column}} $$

$$ a,b \to randomly\;generated\;\;vectors\; of\;\;same\;\; length $$

$$ a_{i} ,b_{i} \in \left\{ {0, \ldots 2^{32} - 1} \right\} $$

$$ m = 2^{32} - 1\;\;\left( {maximum\;\;Hash} \right) $$

$$ p = 2^{61} - 1\;\;({\text{Mersenne prime)}} $$

1 $$ {\text{hmin (}}s_{i} , {\text{a,b) = }}col\_min\left( {\left( {\left( {a \times s_{i}^{T} + b} \right)mod p} \right)mod m} \right) $$

In this work, we investigate twelve different variations of the atom pair MinHashed fingerprint considering different shingle radii *r* as MAP2 (*r* = 1), MAP4 (*r* = 2), MAP6 (*r* = 3), and MAP8 (*r* = 4), each of them in a 1024-dimensions and 2048-dimensions versions, as well as 2048-dimensions folded (instead of MinHashed) variants using the modulo operation in form of foldedAP2 (*r* = 1), foldedAP4 (*r* = 2), foldedAP6 (*r* = 3), and foldedAP8 (*r* = 4).

### Peptide benchmark datasets

Thirty random linear sequences (ten 10-mers, ten 20-mers, and ten 30-mers) were generated with each of all 20 proteogenic amino acids picked with the same probability (Additional file 1: Table S1). For each sequence, we produced 10,000 scrambled unique versions using all amino acids of the parent sequence in random different combination. We also produced 10,000 mutated unique versions by considering the sequence length as the maximum number of possible mutated residues, and for each possible number of point mutations, we generated *n* mutated sequences, where *n *= *ceiling (10,000/maximum number of possible mutations)*; if more than 10,000 sequences were produced, only the first 10,000 were selected. The scrambled and the mutated sets were searched with BLAST [41] using the original sequence as a query. The search was performed with blastp using default settings (Gap opening penalty = 11, Gap extension penalty = 1, Expectation value = 10.0, Word size = 3, Max scores = 25, Max alignments = 15, Query filter = SEG, Matrix = blosum62). The resulting BLAST analogs (Expectation value < 10.0) were labelled as active, while the remaining sequences were labelled as decoys. The protonated SMILES of all peptide sequences were generated using a method of the recently published Peptide Design Genetic Algorithm (PDGA) [28]. To generate the extended fingerprint benchmark training lists for each peptide dataset, 50 different sets of 5 actives and 10% of decoys were randomly picked and stored using the Python package pickle. The peptide active and inactive datasets and the training lists can be found at https://github.com/reymond-group/map4.

### Benchmark metrics and parameters

To evaluate the fingerprints in the extended benchmark, we used the following metrics: AUC, EF1, EF5, BEDROC20, BEDROC100, RIE100, and RIE20. The virtual screening was repeated five times with five different queries. To assess similarity (or dissimilarity) among molecules in the benchmark virtual screenings, we used the Jaccard similarity for MinHash-based fingerprints, Manhattan distance for the 217-dimensions atom-pair fingerprint MXFP (macromolecule extended atom-pair fingerprint), and Dice similarity in all other cases. Details regarding the benchmark implementation can be found in the 2013 Riniker et al. publication [13].

### Similarity search databases preprocessing

ChEMBL 25.0 and Metabolome 4.0 were extracted and manipulated as follows: (1) All structures were canonicalized and chirality information was removed using RDKit; (2) fragments were removed; (3) Heavy atoms were counted using RDKit and compounds with less than 2 heavy atoms were discarded. The filtering resulted in 1,699,888 and 96,456 unique SMILES for the ChEMBL and Metabolome datasets respectively. For ChEMBL molecules, activity information was extracted if present but only when the confidence score was above 5 for a standardized value ≤ 10,000 nM. In the Human Metabolome database preprocessing, the metabolite source was always annotated if available. Natural peptide sequences with 50 of fewer residues were extracted from the SwissProt dataset and translated into non-chiral SMILES using PDGA [28], resulting in 9054 unique structures.

The three datasets were encoded with MAP4 and MHFP6 in 512-dimensions. For each database and fingerprint variant, an LSH forest of 32 trees was generated using the TMAP class. These LSH forests were used as an index for the similarity search. For details on MHFP6, and LSH forest implementation please refer to the recent Probst and Reymond publications [16, 33].

### Similarity search implementation

A fast similarity search tool was implemented for ChEMBL, SwissProt, and the Metabolome databases. The given query is canonicalized and chirality information is removed with RDKit. Then, the nearest neighbors of the processed query are retrieved using the LSH forest corresponding to the chosen database to search in. The query molecule can be provided as a SMILES (drawn structure or pasted SMILES in the JSME editor) [42] or as a linear sequence of a natural peptide. In the latter case, the sequence is transformed into its corresponding SMILES using PDGA as for the SwissProt database and the benchmark compounds. The code of the similarity search is available at https://github.com/reymond-group/map4.

### Databases preprocessing for TMAP

For SwissProt, the previously mentioned similarity search LSH forest was used. ChEMBL 25.0, Metabolome 4.0, and Drugbank 5.4 were extracted and compounds with less than 2 atoms were discarded, resulting in 1,870,343, 114,016, and 10,607 SMILES for the ChEMBL, Metabolome, and Drugbank datasets respectively. A subset of the ChEMBL database was generated by random sampling of 187,034 compounds (10%). Activity information of ChEMBL molecules and sources of metabolome molecules were extracted as previously described for the Similarity Search databases. To provide a TMAP focused on the larger structures in the database, ChEMBL molecules that broke more than one Lipinski’s rules of five [10] were collected to form an additional dataset containing 229,067 entries (Lipinski descriptors were calculated using RDKit).

For the SwissProt database, positive and negative charges were calculated directly from the peptide sequences: R and K counted as a positive charge each, D and E counted as a negative charge each, all other residues were considered neutral. The number of aromatic atoms (AR) was calculated counting all lowercase “c”, “n”, “s”, and “o” not belonging to a two-letter element in the canonical SMILES. All other properties were calculated using RDKit.

The five datasets were encoded with MAP4 in 512-dimensions. For each database and fingerprint version, an LSH forest of 32 trees was generated using the TMAP class. The obtained LSH forests were used to layout the corresponding TMAPs. The color-codes of property values on each TMAP (accessible via the TMAP menu) were obtained by first ranking molecules using SciPy [43], and then assigning the rank to a color linearly along the color scale. For the property “Phosphorus count” we used a *dense* ranking, in which molecules with the same number of P atoms receive the same rank. For all other properties a standard (or *average*) ranking was used: the average of the ranks that would have been assigned to all the tied values was assigned to each value. For details on TMAP please refer to the related publication [33].

### Nearest neighbor analysis

The Human Metabolome data set was sorted unique after removing stereochemistry information and for each molecule, the distance from its nearest neighbor was calculated in the MAP4-1024, MHFP6-1024, TT (not hashed), AP (not hashed), and ECFP4-1024 chemical spaces. AP, TT, and ECFP4 were calculated with RDKit. In each fingerprint space, for each structure, a similarity search against the entire dataset was performed and the NN retrieved. The similarity was assessed as Tanimoto Distance calculated with RDKit.

## Results and discussion

### Fingerprint design

Our atom-pair fingerprint is designed similarly to the AP fingerprint implemented by RDkit. AP encodes atom pairs using atomic invariants combined with their bond distances. Instead of using atomic invariants, we use the circular environment of each atom in the pair up to a preset radius, written as canonical SMILES, similar to the method used for MHFP6. Recording circular substructures is expected to lead to a more detailed perception of substructures in the fingerprint enabling better performance in small molecule benchmarks, while the bond distance information should translate into a perception of molecular size and shape. For each radius value *r* (typically *r* = 1 and 2), we encode each atom pair as a character string consisting of the two canonical SMILES of the circular substructure around each atom up to the set radius and the bond distance information. We then hash these atom-pair strings and use MinHash to produce the actual fingerprint to capitalize on the advantages of this approach over binary encoding as previously demonstrated with MHFP6 (see “Methods”, Eq. 1) [16]. For example, our MinHashed Atom Pair fingerprint with *r* = 2 (MAP4) encodes pairs of circular substructures with radius *r* = 1 and 2 (Fig. 1).

### Benchmarking study design

To evaluate the performance of MAP4 we use a modified version of the fingerprint benchmark developed by Riniker and Landrum [13]. The benchmark provides a detailed insight about the performance of an evaluated fingerprint in the recovery of actives in a virtual screening of a database of known actives and decoys, where the actives/decoys sets are taken from the DUD [44], the MUV [45], and the ChEMBL [35] datasets. However, since most molecules are within the rules of five limits (Additional file 1: Figure S1), the benchmark gives no explicit information on the performance of an evaluated fingerprint in encoding larger molecules. We have therefore extended the benchmark with a series of peptides as exemplary large biomolecules not only because they are an important class of drugs, but also because their similarity can be assessed with BLAST, a reliable and widely used tool. Our peptide benchmark consists of 60 scrambled and mutated peptide datasets generated from 30 randomly generated sequences. In each set the actives and decoys are defined through their sequence similarity to the corresponded query: the BLAST analogs are labelled as active, while the remaining sequences are labelled as inactive (see “Methods” and Table 1).

**Table 1:** Average number and percentage of actives in all datasets used for the benchmark

	MUV^a^	DUD^a^	ChEMBL^a^	Mutated peptides^b^	Scrambled peptides^b^
Average n.o. actives	30.0 ± 0.0	91.3 ± 80.5	100.0 ± 0.0	500.2 ± 0.7	56.0 ± 27.4
Average % actives	0.2 ± 0.0%	2.2 ± 0.4%	1.0 ± 0.0%	5.3 ± 0.0%	0.6 ± 0.2%

^a^Known actives used in the Riniker and Landrum [13] benchmark

^b^BLAST analogs of a defined query generated for this study

We include 21 different fingerprints in the comparison, comprising the 12 variations of our MAP4 fingerprint as described in the “Methods”, and nine reference fingerprints performing particularly well for small or large molecules. This reference set includes ECFP4 and MHFP6 in their 1024-dimensions and 2048-dimensions versions as best performing fingerprints for small molecules, MXFP (macromolecule extended atom-pair fingerprint, 217-dimensions atom-pair fingerprint) as a good performing fingerprint for large molecules and peptides [27, 28], and the Atom Pair (AP) and Topological Torsion (TT) fingerprints from RDKit. In the AP and TT fingerprints atoms are represented using their atom type, their number of heavy neighbors, and their number of pi electrons. AP encodes all atom pairs and their distance as a number, while TT encodes all atoms along the path between two atoms up to topological distance of four bonds. Note that AP and TT are not hashed as in the original benchmark. Finally, our reference set includes MACCS and ECFP0 as baseline fingerprints following the Riniker benchmark [13].

**Fig. 2:**
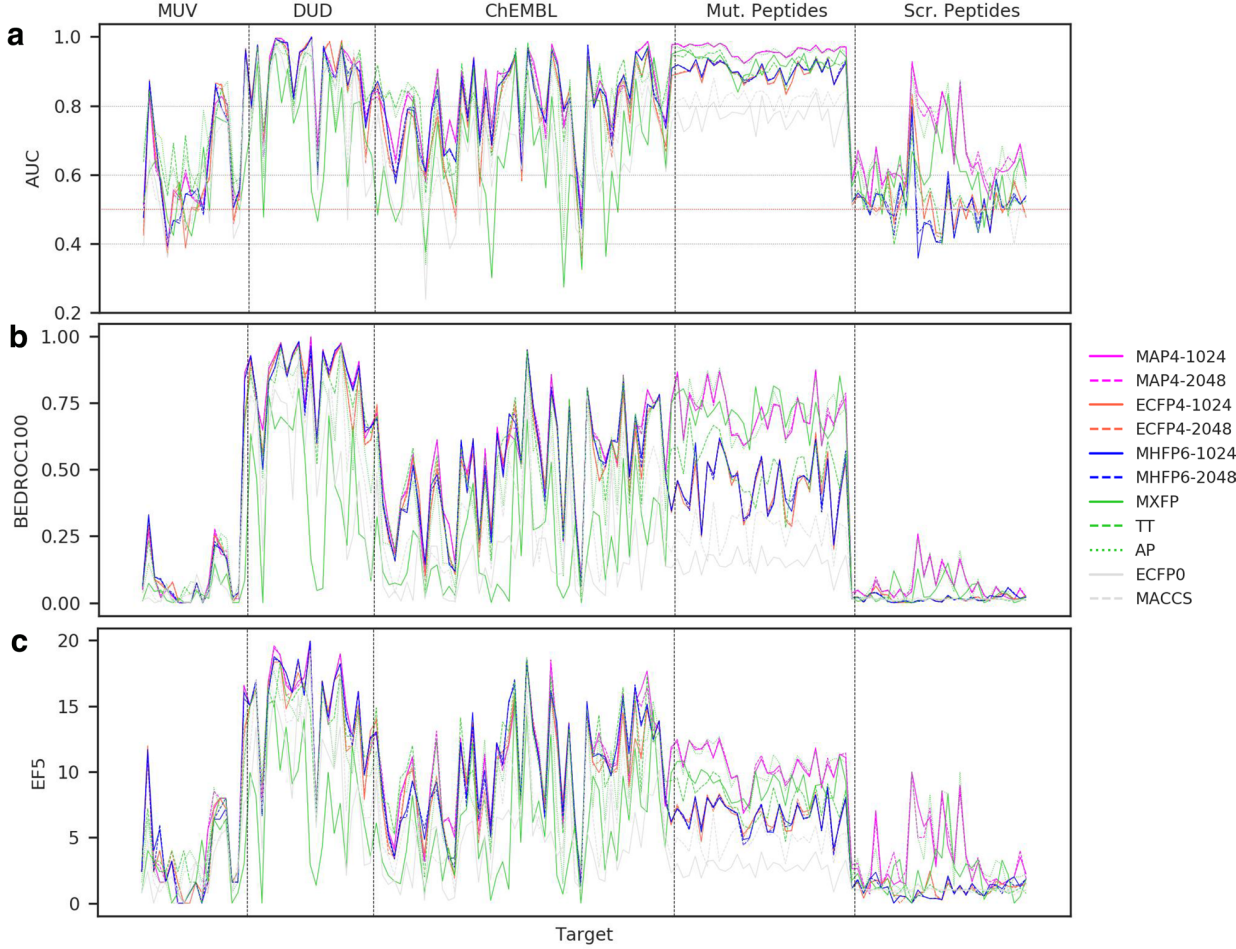
AUC (**a**), BEDROC100 (**b**), and EF5 (**c**) of MAP4 (magenta), ECFP4 (orange), MHFP6 (blue), MXFP (solid green line), TT (dashed green line), AP (dotted green line), MACCS (solid gray line), and ECFP0 (dashed gray line) across all small molecules and peptide targets (17 MUV targets, 21 DUD targets, 50 ChEMBL targets, 30 mutated peptide targets, and 30 scrambled peptide targets)

**Fig. 3:**
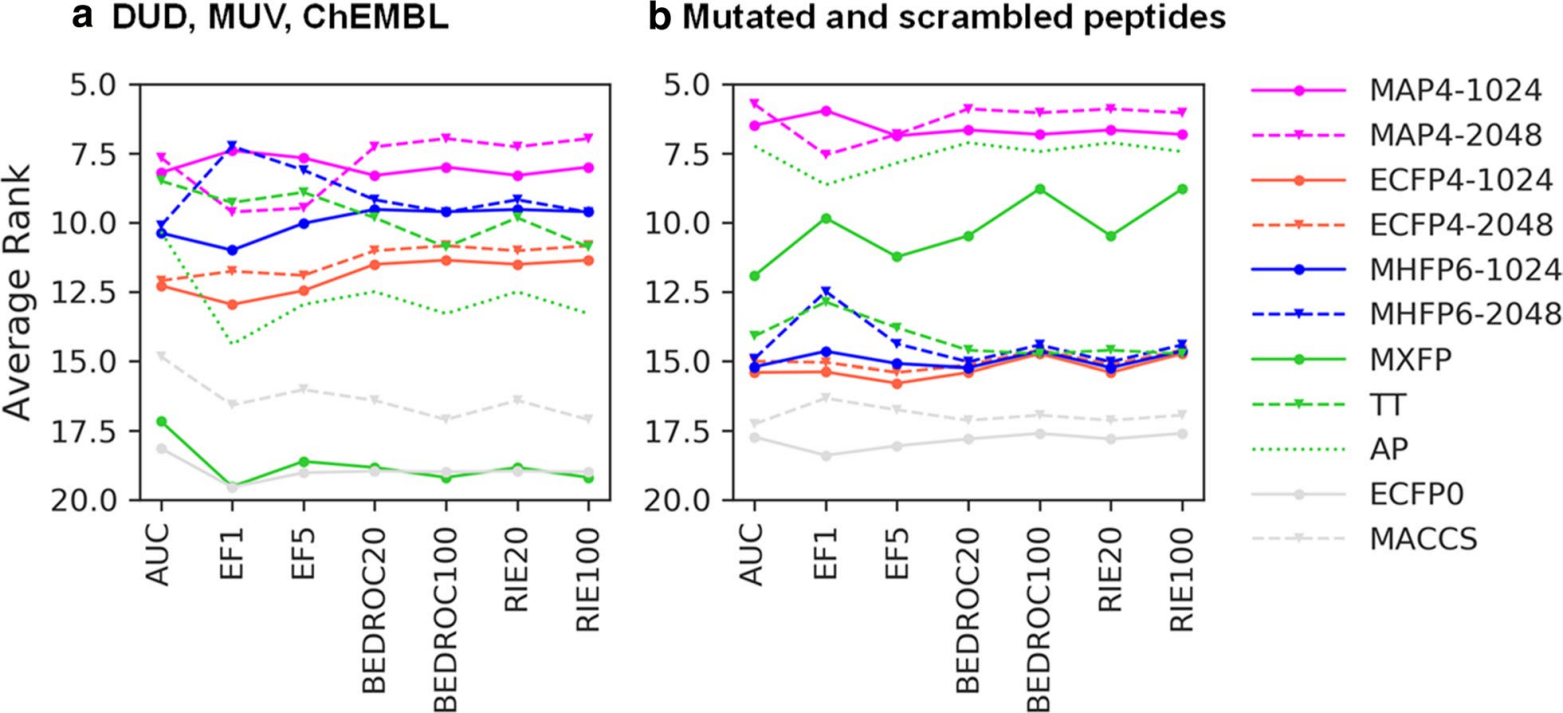
Average ranking of MAP4 (magenta), ECFP4 (orange), MHFP6 (blue), MXFP (solid green line), TT (dashed green line), AP (dotted green line), MACCS (solid gray line), and ECFP0 (dashed gray line) in in the fingerprint benchmark when using only small molecules datasets (17 MUV targets, 21 DUD targets, and 50 ChEMBL targets, **a** and only peptide datasets (30 mutated peptide targets and 30 scrambled peptide targets, **b** note that 11 out of 21 fingerprints are shown

We use five different metrics in the benchmark, namely AUC (Fig. 2a), RIE100 (Additional file 1: Figure S2a) and RIE20 (Additional file 1: Figure S2b), BEDROC100 (Fig. 2b) and BEDROC20 (Additional file 1: Figure S2c), and EF1 (Additional file 1: Figure S2d) and EF5 (Fig. 2c). The relative performance of the different fingerprints is then assessed by computing their average rank in each of the metrics following the Riniker approach (Fig. 3a–c). The statistical relevance of the ranks is assessed with the Friedman Test provided in the Riniker benchmark, where the post hoc analysis is performed using Wilcoxon-Nemenyi-McDonald-Thompson test (Additional file 1: Figures S3–S5) [46, 47].

### Benchmarking results

We first compare MAP4 with the nine reference fingerprints presented above. In the small molecule benchmark MAP4 is slightly better than substructure fingerprints (ECFP4, MHFP6, and TT), yet the difference is not statistically significant. However, MAP4 outperforms atom-pair fingerprints such as AP and MXFP, which perform significantly worse in this benchmark (Fig. 3a and Additional file 1: Figure S3). The situation is reversed in the peptide benchmark, where atom-pair fingerprints significantly outperform substructure fingerprints (Fig. 3b). MAP4 performs best among these atom-pair fingerprints, however, the difference is not statistically significant (Additional file 1: Figure S4). Remarkably, MAP4 is the only fingerprint maintaining good performances in both benchmarks.

**Fig. 4:**
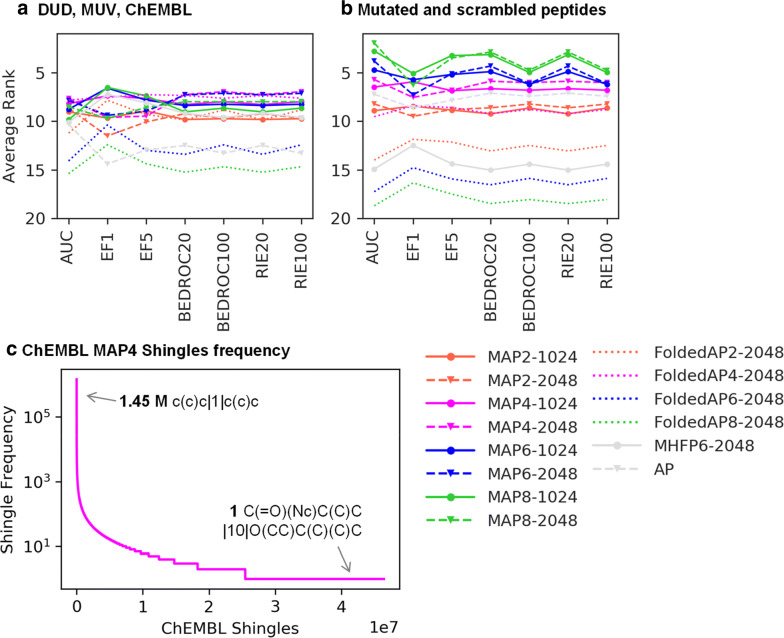
**a**, **b** Average rank of AP2 (orange), AP4 (magenta), AP6 (blue), AP8 (green), in their 1024-dimensions (solid) and 2048-dimensions (dashed) MinHashed implementation (MAPs), and in their 2048-dimensions folded (dotted) implementation (foldedAPs) in the fingerprint benchmark when using only small molecules datasets (17 MUV targets, 21 DUD targets, and 50 ChEMBL targets, **a**) and only peptide datasets (30 mutated peptide targets and 30 scrambled peptide targets, **b**). In both panels **a** and **b**, MHFP6 (solid) and AP (dashed) are reported in grey. Note that 14 out of 21 fingerprints are shown. **c** ChEMBL MAP4 shingles frequency analysis, examples of shingles with different frequencies are reported

Having established that MAP4 outperforms other known fingerprints in the combined small molecules and peptides tasks, we next investigate if further improvements might be possible in 12 variations of the MAP4 fingerprint considering different shingle radii (*r* = 1, 2, 3, 4), compression methods (MinHash versus folding), and the number of dimensions (1024 or 2048). We include MHFP6-2048 and the RDKit AP as reference fingerprints in this comparison. Comparing the average fingerprint rank for small molecules (Fig. 4a) and peptides (Fig. 4b), as well as the performance metrics on each dataset (Additional file 1: Figure S5) shows that the MinHashed fingerprints (MAPs) rank better than their folded versions (foldedAPs) in a statistically significant manner, except for foldedAP2 when using only the small molecule datasets (Additional file 1: Figures S3, S4). The better performance of MinHashed over folded versions of the same fingerprint was already observed in our study of MHFP6 [16], and probably results from the fact that MinHashing creates fewer unintended bit collisions as compared to modulo-based hashing (folding) as an information compression method. Bit collision is most likely also the reason for the decreasing performance of foldedAPs when the radius, and therefore the encoded information, increases.

Among the different MAPs, those with larger radii perform better, however, the difference is not statistically significant. At the same time increasing the radius from *r* = 1 (MAP2) to *r* = 2 (MAP4), *r* = 3 (MAP6) and *r* = 4 (MAP8) defines an exponentially increasing number of unique atom-paired molecular shingles, as exemplified for the case of the ChEMBL database (Table 2). The selected MAP4 (*r* = 2) represents a compromise to represent substructures in reasonable but not exaggerated detail. In the MAP4 ChEMBL space, there are 46,430,912 atom-pair molecular shingles. While half of them are seen only once, the most common Shingle is present in 85% of ChEMBL structures (Fig. 4d). Note that the radius can be selected by the user in the current implementation.

**Table 2:** Analysis of ChEMBL using MinHashed atom-pair fingerprint variants

Fingerprint^a^	Unique shingles^b^
MAP2 (*r* = 1)	1,913,607
MAP4 (*r* = 2)	46,430,912
MAP6 (*r* = 3)	205,576,613
MAP8 (*r* = 4)	465,393,948

^a^MinHashed atom-pair fingerprint version with different shingle radii

^b^Number of different atom-paired molecular shingles in the entire ChEMBL database

The above benchmarking study shows that our MinHashed Atom-Pair fingerprint MAP4 performs among the best fingerprints for small molecules and the best fingerprints for peptides, but is the only fingerprint performing best on both benchmarks. We attribute this combined performance to the fact that MAP4 combines circular substructures, which are optimal to describe small molecules, with atom pairs as a method particularly well suited for large molecules. The benchmark among the different MAP versions furthermore shows that the level of detail perceived by the 1024-dimensions MAP4 version is optimal for good performance.

### Chemical space maps

To further illustrate the suitability of MAP4 as a molecular fingerprint across various molecule families, we consider different databases covering various molecular size ranges and types (Table 3), and visualize them in form of chemical space tree-maps (TMAPs) [33]. These interactive tools can be readily computed exploiting the fact that similarly to MHFP6, MAP4 is a MinHashed fingerprint, for which one can use locality sensitive hashing (LSH) for computing the k-NN tree that is represented in the TMAP even for databases of millions of molecules. The TMAPs discussed below are freely accessible at http://tm.gdb.tools/map4/.

**Table 3:** Databases illustrated as MAP4 tree-maps

Database	Size^a^	HAC^b^
ChEMBL^c^	1,870,343	30.0 ± 17.5
Non-Lipinski ChEMBL	203,850	55.7 ± 38.7
Human metabolome	114,016	61.7 ± 28.1
SwissProt	9054	237.4 ± 104.7
DrugBank	229,067	26.2 ± 20.7

^a^Number of molecules in the database after pre-processing (see “Methods”)

^b^HAC = heavy atom count given with standard deviation. All non-hydrogen atoms in the molecule

^c^The TMAP for ChEMBL is limited to a random 10% subset (187,034 compounds) to reduce server load

**Fig. 5:**
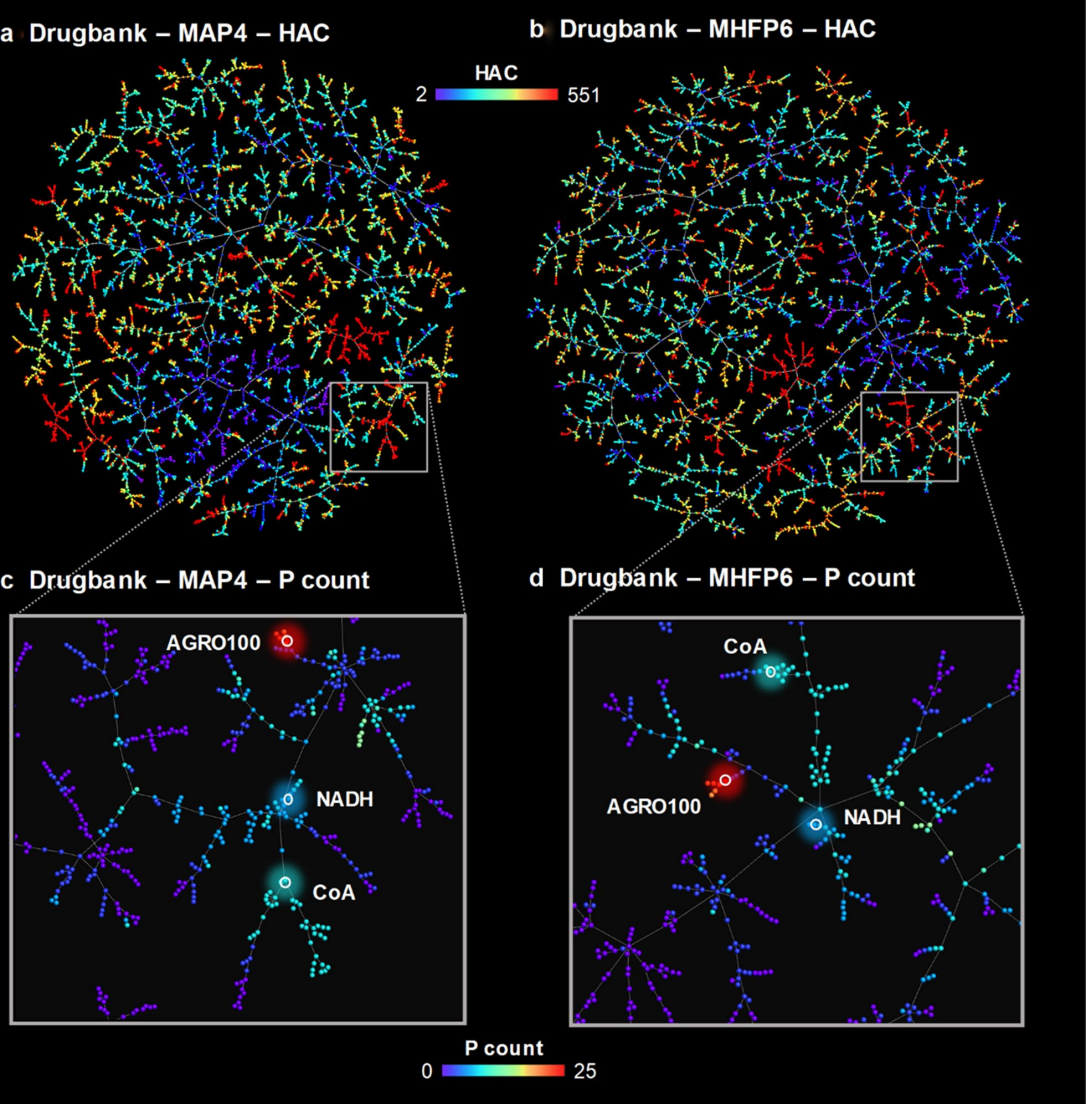
TMAPs of Drugbank using MAP4 and MHFP6. **a** MAP4 TMAP color-coded by molecule size (HAC). **b** MHFP6 TMAP color-coded by molecule size. (**c**) Close-up view of **a** color-coded by the number of phosphorous atoms per molecule (P count). **d** Close-up view of **b** color-coded by P count. Interactive TMAPs of Drugbank, ChEMBL, and non-Lipinski ChEMBL, color-coded with additional properties, are accessible at http://tm.gdb.tools/map4/

**Fig. 6:**
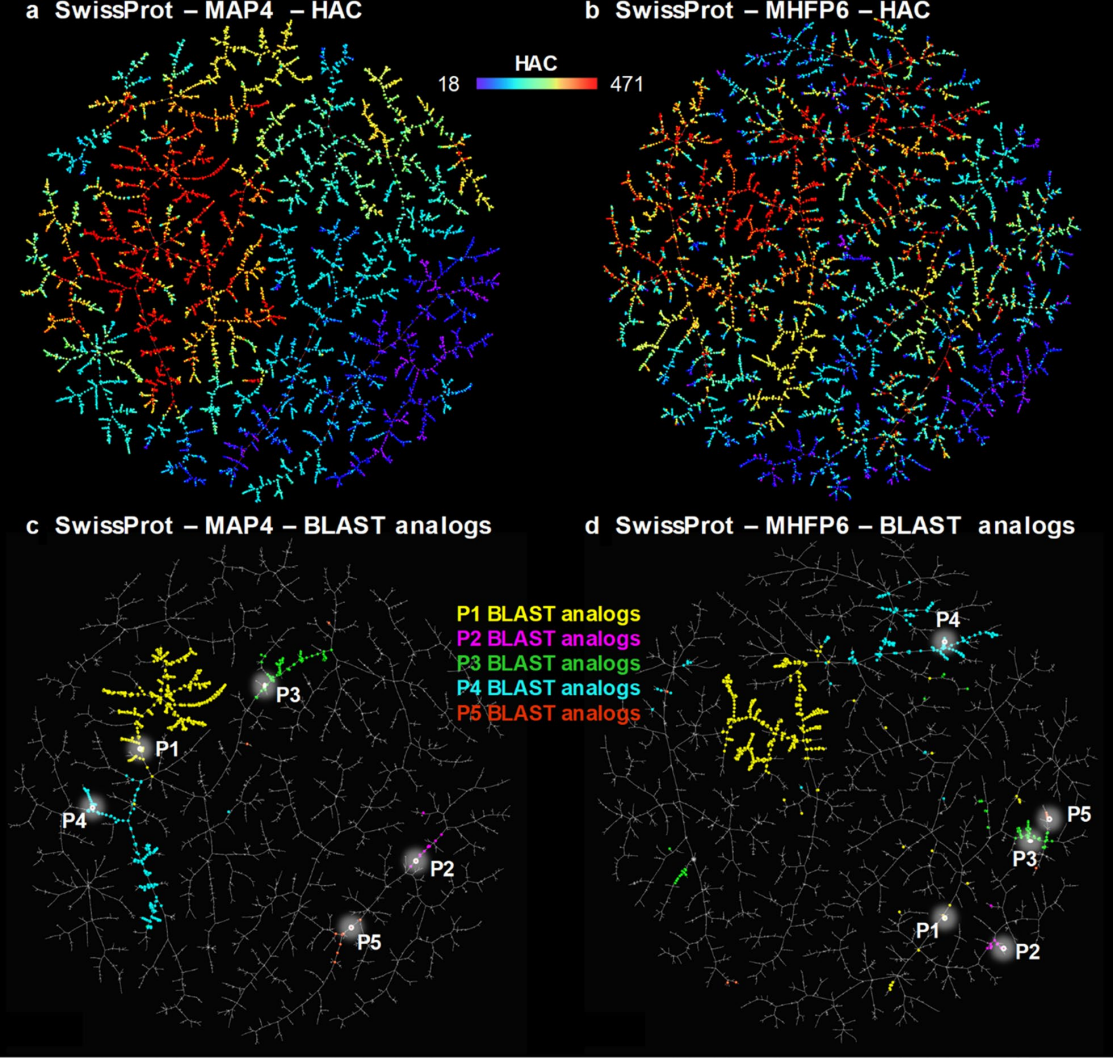
TMAPs of the SwissProt dataset. **a** MAP4 TMAP and **b** MHFP6 TMAP color-coded by HAC. **c** MAP4 TMAP and **d** MHFP6 TMAP color-coded by BLAST analogs of MTQRTLRGTNRRRIRVSGFRARMRTASGRQVLRRRRAKGRYRLAVS (P1), MELFAALNLEPIFQLTFVALIMLAGPFVIFLLAFRGGDL (P2), TNRNFLRF (P3), and MRVNITLECTSCKERNYLTNKNKRNNPDRLEKQKYCPRERKVTLHRETK (P4), INLKALAALAKKIL (P5) in the MAP4 (**c**) and MHFP6 (**d**) chemical spaces. The interactive maps are accessible at http://tm.gdb.tools/map4/

Comparing MHFP6 and MAP4-based TMAPs for the ChEMBL database [35], its non-Lipinski subset [27], and DrugBank [34] shows that both fingerprints perform comparably well in organizing these databases. Although one would expect that MAP4 would perform better than MHFP6 in separating molecules by size, this is not the case (Fig. 5a, b). The ability of MHFP6 to separate molecules by size reflects the fact that in these databases, large molecules contain either a larger diversity of substructures or simply different substructures compared to small molecules, which results in an implicit size perception in the substructure encoding even if these substructures are small. The ability of both MAP4 and MHFP6 to classify molecules across different size ranges is well illustrated by visualizing phosphorous-containing molecules, which span from inorganic phosphates through cofactors (CoA, NADH) to large therapeutic oligonucleotides (AGRO100, Fig. 5c, d). On the other hand, in TMAPs of the SwissProt dataset MAP4 separates molecules by size much better than MHFP6 (Fig. 6a, b). In this case BLAST analogs are also better grouped in the MAP4-based maps than in the MHFP6-based maps, in line with the peptide benchmark study (Fig. 6c, d).

**Fig. 7:**
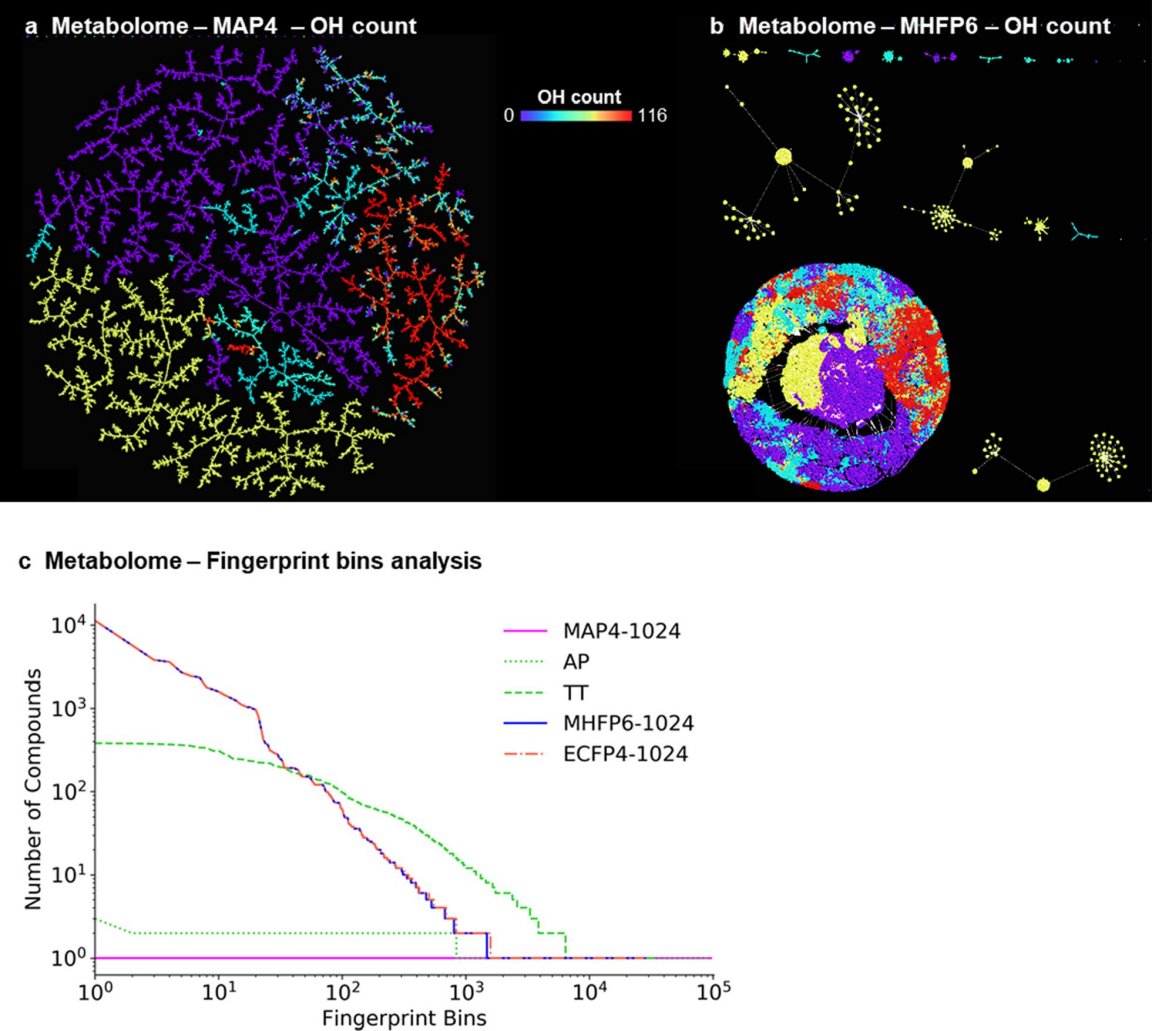
TMAPs of the Human Metabolome Database. **a** MAP4 TMAP and **b** MHFP6 TMAP color-coded by OH count. The interactive maps with additional properties are accessible at http://tm.gdb.tools/map4/. **c** Human Metabolome compounds per fingerprint bins in the MAP4-1024 (magenta line, solid), AP (green line, dashed), TT (green line, dotted), MHFP6-1024 (blue line, solid), and ECFP4-1024 (orange line, dash-dotted) chemical spaces

MAP4 also performs much better than MHFP6 for mapping the Human Metabolome Database (HMDB). This database contains diverse lipids, phospholipids, carbohydrates, glycosides, amino acid derivatives and more [38]. In this case, MAP4 produces a very well defined TMAP because encoding atom-pairs up to any distance ensures a differentiation between molecules containing different numbers of repetitive substructures such as lipids and glycosides (Fig. 7a). By contrast, MHFP6 fails to properly distinguish between related metabolites and the map consists of very large groups of molecules appearing as “grapes” (Fig. 7b). Analyzing the occupancy of fingerprint value bins shows that for the three substructure fingerprints, the ten most populated fingerprint value bins contain a large number of molecules, thousands for ECFP4 and MHFP6 and hundreds for TT (Fig. 7c). These molecules are lipids and phospholipids, and in the case of ECFP4 and MHFP6, these are the same molecules (Additional file 1: Figures S6–S8). By contrast, atom-pair fingerprints contain either a single molecule per bin (MAP4) or at most two or three molecules per bin (AP).

### Nearest neighbor searches

The difference in the MAP4- and MHFP6-based TMAPs of HMDB reflects the ability of MAP4 to distinguish between closely related metabolites perceived as identical by MHFP6. HMDB contains 96,456 structurally different metabolites not considering stereochemistry. Performing an exhaustive nearest-neighbor (NN) search on these metabolites shows that MAP4 distinguishes all metabolites from one another without exception (Table 4). By contrast MHFP6 finds an indistinguishable NN (JD = 0) in 72.5% of HMDB molecules. The situation is even slightly worse with ECFP4 (72.9%) and slightly better with TT (71.1%). On the other hand, AP sees an indistinguishable NN in only 1677 molecules (1.7%) and is therefore almost as good as MAP4.

**Table 4:** Nearest neighbor analysis of the human metabolome database

HMBD subset	All	OH = 0	OH = 1	1 < OH ≤ 4	OH > 4
All	96,456	33,721	10,663	41,493	10,579
JD (MAP4-1024) = 0	0	0	0	0	0
JD (AP) = 0	1677	13	35	1611	18
JD (TT) = 0	68,623	27,897	5782	32,909	2035
JD (MHFP6-1024) = 0	69,972	28,502	6215	33,359	1996
JD (ECFP4-1024) = 0	70,329	28,561	6243	33,294	2231

Subsets of the Human Metabolome 4.0 Database according to the number of hydroxyl groups per molecule separating lipids (OH = 0, 1) from carbohydrate derivatives (OH > 4). For each subset (column), the number of molecules is indicated in total (All, line 2) and counting those with an indistinguishable nearest neighbor (Jaccard Distance JD = 0) according to the indicated fingerprint (line 3–7). Molecules were considered after removing stereochemical information

**Fig. 8:**
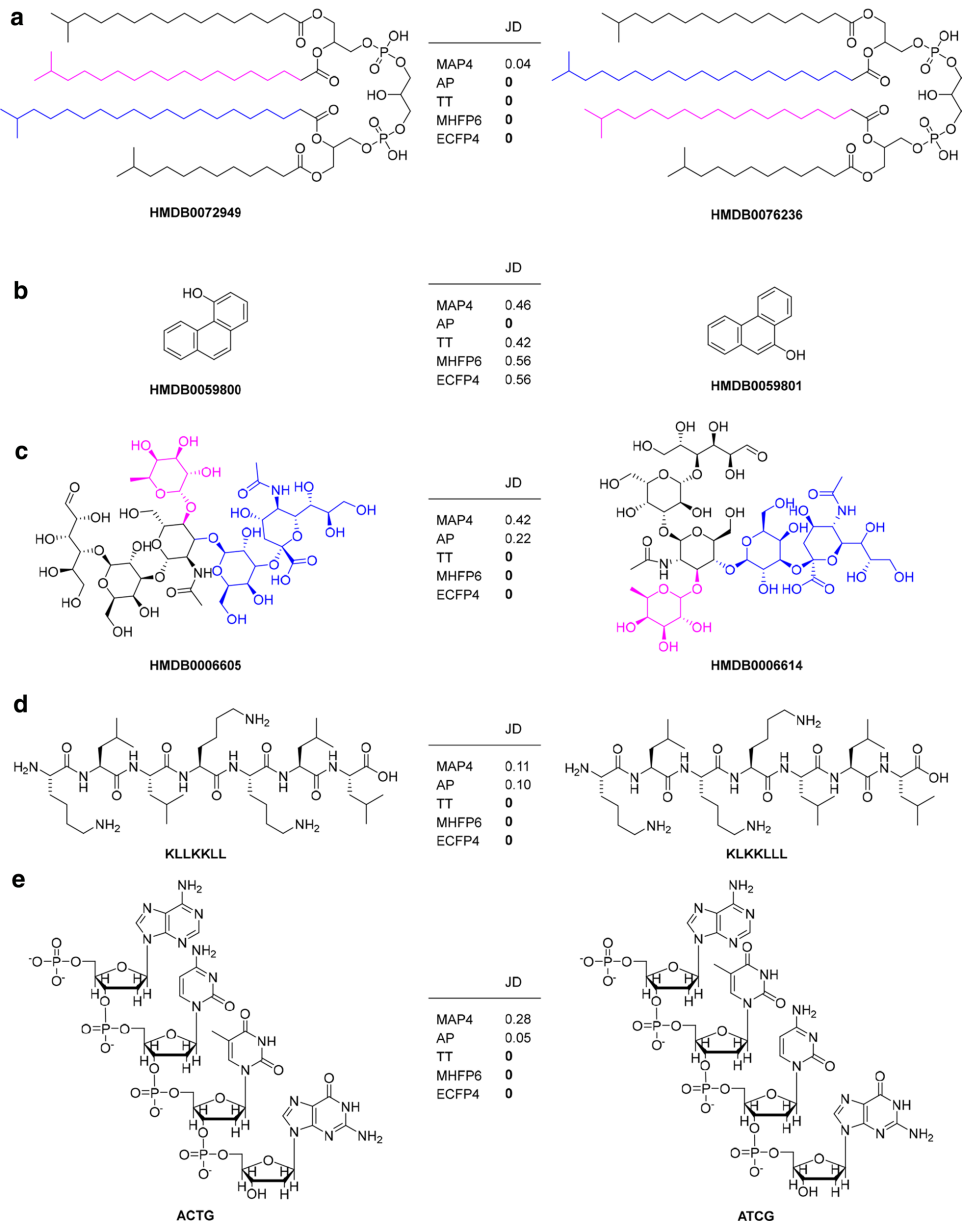
Pairs of molecules better differentiated with MAP4 than with MHFP6, MAP4, TT, AP, and ECFP4 and their JD values. **a** Lipids from HMDB, the different position of the lipidic chains is highlighted using blue and magenta. **b** Phenanthrol isomers from HMDB. **c** Hexasaccharides from HMDB, the α-l-fucosyl and β(3-sialyl)-galactosyl groups exchanged at positions 3 and 4 of the central *N*-acetylglucosamine are highlighted using blue and magenta (structures as given in HMDB with open-chain form of the first carbohydrate and missing stereochemistry at one center each). **d** Scrambled heptapeptides. **e** Scrambled tetranucleotides

HMDB can be sorted by OH-count, which approximately separates triglycerides and related apolar lipids (OH = 0), diglycerides, alcohols and acids (OH = 1), phospholipids (1 < OH ≤ 4) and carbohydrates (OH > 4). Analyzing the number of indistinguishable NN as a function of OH count shows that AP mostly fails with phospholipid-type molecules (1 < OH ≤ 4), where 96.1% of the 1677 AP-indistinguishable NN are found. A remarkable example is provided by the complex phospholipids HMDB0072949 and HMDB0076236, which are distinguished from one another only by MAP4 (Fig. 8a). AP also fails to distinguish between 4-phenanthrol (HMDB0059800) and 9-phenanthrol (HMDB0059801), the latter being an inhibitor of the ion channel TRPM4 (Fig. 8b) [48]. This lack of differentiation by AP is somewhat surprising since all other fingerprints easily distinguish between these two isomers, and reflects the fact that AP is the only fingerprint in the series which does not perceive atom environments but only atomic properties.

MAP4 and AP perceive differences between many closely related metabolites that are indistinguishable for substructure fingerprints. An interesting example among carbohydrates is provided by the branched hexasaccharides HMDB0006605 and HMDB0006614, which only differ from one another by the permutation of the fucoside and 4-sialyl-galactoside at the C(3)-OH and C(4)-OH groups of the central *N*-acetylglucosamine (Fig. 8c). This differentiation is enabled by the encoding of atom-pairs at distances longer than the maximum length spanned by the substructure fingerprints MHFP6 (six bonds), ECFP4 and TT (four bonds).

Encoding atom-pairs at long distances is also what enables atom-pair fingerprints to perform well in the peptide benchmark discussed above where BLAST-analogs must be recovered from scrambled or mutated sequences. This is well illustrated for NN searches in the case of heptapeptides KLLKKLL and KLKKLLL, which are only distinguished from one another by MAP4 and AP (Fig. 8d). A similar situation arises when considering oligonucleotides such as the pair ACTG and ATCG which only differ by the permutation of the two central pyrimidine bases (Fig. 8e).

Inspecting nearest neighbors of any molecule of interest provides an additional opportunity to explore the content of large databases, often as a means to perform virtual screening to identify analogs. The MinHashed nature of MAP4 enables us to perform extremely rapid approximate nearest neighbor (k-NN) searching using locality sensitive hashing (LSH). We have therefore prepared MAP4 similarity search portals for the ChEMBL, the Human Metabolome, and the SwissProt subset described above, which are freely accessible at http://map-search.gdb.tools/. Note that NN-searches using LSH forests are approximate and not identical with the exact NN-searches using in the benchmarking study, however, it is well-known that the results of approximate k-NN searches based on LSH forests are not significantly different from exact k-NN searches [49].

## Conclusion

In summary, combining the principles of circular substructures, atom-pairs, and MinHashing produces the MinHashed atom-pair fingerprint MAP4. MAP4 is a new molecular fingerprint performing as good as extended connectivity fingerprints such as ECFP4 and MHFP6 on the Riniker and Landrum small molecule benchmark, and as good as the RDkit AP fingerprint on a new peptide sequence similarity benchmarking set for recovering BLAST analogs among scrambled and mutated peptide sequences, designed to evaluate performance on large molecules. The high performance of MAP4 in the small molecule benchmark is made possible by the substructure encoding which is absent in previous atom-pair fingerprints, while high performance in the peptide benchmark reflects the perception of atom-pairs at unrestricted topological distances which is missing in substructure fingerprints. While the current version of the MAP fingerprint is implemented in Python and therefore it is relatively slow, the performance might increase by rewriting the fingerprint in C or C ++.

The MinHashing used for MAP4 allows the construction of k-NN trees and the creation of high-resolution chemical space tree-maps (TMAPs) for databases as diverse as DrugBank, ChEMBL, Swissprot, and the Human Metabolome. The MAP4 based TMAPs are much better defined than those obtained using the substructure MinHashed fingerprint MHFP6, in particular for the case of the Human Metabolome. This is because MAP4 perceives differences among highly similar molecules such as lipids with related fatty acid chains which are not seen by MHFP6. MAP4 also distinguishes between high-similarity pairs of peptides and oligonucleotides perceived as identical by substructure fingerprints such as MHFP6. MAP4 represents a universal fingerprint to search and map the chemical space across molecules of all types and sizes and should be generally useful in the field of cheminformatics.

## Supplementary information

**Additional file 1: Table S1.** Linear random peptide sequences used to generate the mutated and scrambled peptide datasets for the extended fingerprint benchmark. **Figure S1.** Hydrogen bond acceptor and donor count, Molecular Weight, and calculated logarithm octanol–water partition coefficient of the actives/decoys used in the original version of the Riniker fingerprint benchmark. **Figure S2.** RIE100, RIE20, BEDROC20, and EF1 of MAP4, ECFP4, MHFP6, MXFP, TT, AP, MACCS, and ECFP0 across all small molecules and peptide targets. **Figure S3.** Relative ranking and p-values of and MAP4-1024 in the Riniker fingerprint benchmark with small molecules datasets. **Figure S4.** Relative ranking and p-values of and MAP4-1024 in the Riniker fingerprint benchmark with peptide datasets. **Figure S5.** AUC, BEDROC100 and 20, EF1 and 5, RIE100 and 20 of MAP4 variants. **Figures S6**–**S8.** Examples of molecules from HMDB found in highly populated fingerprint bins for ECFP4, MHFP6, and TT.

## Data Availability

The code for the MAP4 fingerprint is available at https://github.com/reymond-group/map4. Interactive MAP4 similarity search tools and TMAPs for various databases are accessible at http://map-search.gdb.tools/ and http://tm.gdb.tools/map4/.

## Abbreviations

AP: Atom pair fingerprint
AUC: Area under the curve
BEDROC: Boltzmann-enhanced discrimination of the receiver operating characteristic
BLAST: Basic local alignment search tool
DUD: Directory of useful decoys
CoA: Coenzyme A
CS: Circular substructure
ECFP: Extended connectivity fingerprint
EF: Enrichment factor
HAC: Heavy atom count
HMDB: Human metabolome database
JD: Jaccard distance
LSH: Locality sensitive hashing
MAP: MinHash atom pair fingerprint
MHFP: MinHash fingerprint
MUV: Maximum unbiased validation data sets
MXFP: Macromolecule extended atom pair fingerprint
NAD: Nicotinamide adenine dinucleotide
NLC: Non-Lipinski ChEMBL
NN: Nearest neighbor
OH: Hydroxy group
RIE: Robust initial enhancement
ROC: Receiver operating characteristic
SMILES: Simplified molecular-input line-entry system
TMAP: Tree map
TP: Topological distance
TT: Topological torsion fingerprint
P: Phosphorus
