# Supplementary material for: One molecular fingerprint to rule them all: drugs, biomolecules, and the metabolome
Source: J Cheminform. 2020 Jun 12;12:43. doi: 10.1186/s13321-020-00445-4 (PMC7291580; doi:10.1186/s13321-020-00445-4)
Supplement: Supplementary file 1 — Additional file 1: Table S1. Linear random peptide sequences used to generate the mutated and scrambled peptide datasets for the extended fingerprint benchmark. Figure S1. Hydrogen bond acceptor and donor count, Molecular Weight, and calculated logarithm octanol–water partition coefficient of the actives/decoys used in the original version of the Riniker fingerprint benchmark. Figure S2. RIE100, RIE20, BEDROC20, and EF1 of MAP4, ECFP4, MHFP6, MXFP, TT, AP, MACCS, and ECFP0 across all small molecules and peptide targets. Figure S3. Relative ranking and p-values of and MAP4-1024 in the Riniker fingerprint benchmark with small molecules datasets. Figure S4. Relative ranking and p-values of and MAP4-1024 in the Riniker fingerprint benchmark with peptide datasets. Figure S5. AUC, BEDROC100 and 20, EF1 and 5, RIE100 and 20 of MAP4 variants. Figures S6–S8. Examples of molecules from HMDB found in highly populated fingerprint bins for ECFP4, MHFP6, and TT. [file 13321_2020_445_MOESM1_ESM.pdf]

Additional file 1

One molecular fingerprint to rule them all: drugs, biomolecules, and the metabolome

Alice Capecchi, Daniel Probst, and Jean-Louis Reymond*

Department of Chemistry and Biochemistry, University of Bern, Freiestrasse 3, 3012 Bern, Switzerland

E-mail: jean-louis.reymond@dcb.unibe.ch

Table S1. Linear random peptide sequences used to generate the mutated and scrambled peptide datasets (available at https://github.com/reymond-group/map4) for the extended fingerprint benchmark.

Length	Random sequences
10 residues	KAQIDLSPNP
	TITVVSLMNQ
	SSQHVQRENY
	PNWKLRPNYH
	QHVEYEQHDL
	LQKLNDTWCT
	HRIWAWVNMN
	VNWVWDHFSR
	IDIIIRTHES
	LFCCVAYSFD
20 residues	NLCADQWYFSNAFW
	VIHGPWLIQGISHAI
	DHVRWIFWHAAICCD
	RTVSFSYCRQDCQPF
	KGHYERMCEPVRKKN
	NNYYPKYREPQGKII
	VGPYVKHPAEACNQQ
	QETTHQIPFMTAFAH
	RYAWTRHFAIFVVDV
	FGDWRAGYGGSPENL
30 residues	IRCWIWVECLYINHRVHEYW
	HQDAICAETWIVKWATLNSW
	HWYEKCCHGAQSWWTVDQWL
	RWPWKPFHVRFFFQRWPLLH
	FPGFIAKCTQWGAGLEVGGH
	SPYDQEMDQQFDRACCWHFK
	VSNWRLLGPRPYMSNTETQR
	NAPSYKTFEANITTRAARNS
	VPMMTDGIMTVYVGGEPWSR
	HAETEGYKSSPTKLGCDPLY

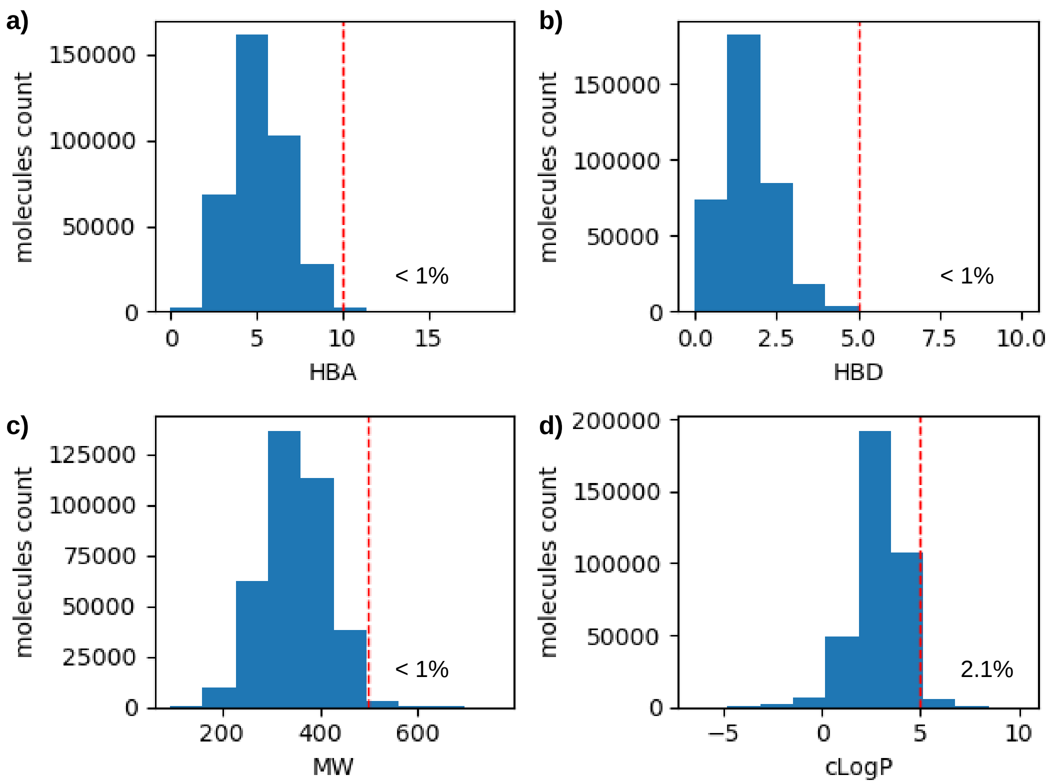

Figure S1. Hydrogen bond acceptor (HBA, a) and donor (HBD, b) count, Molecular Weight (MW, c), and calculated logarithm octanol-water partition coefficient (cLogP, d) of the actives/decoys used in the original version of the Riniker fingerprint benchmark. The percentage of molecules that violate each rule is reported in the corresponded panel.

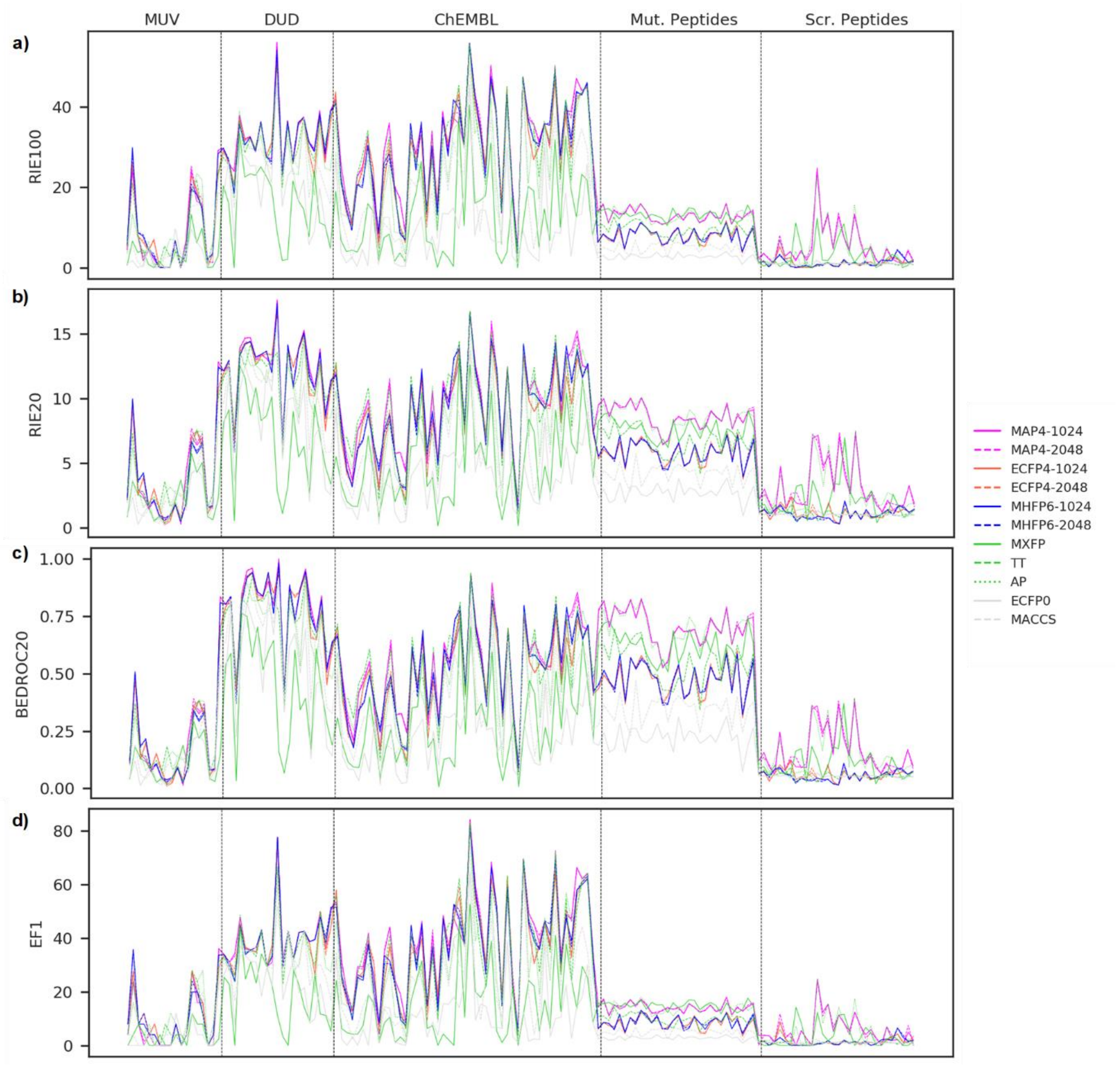

Figure S2. RIE100 (a), RIE20 (b), BEDROC20 (c), and EF1 (d) of MAP4 (magenta), ECFP4 (orange), MHFP6 (blue), MXFP (solid green line), TT (dashed green line), AP (dotted green line), MACCS (solid gray line), and ECFP0 (dashed gray line) across all small molecules and peptide targets.

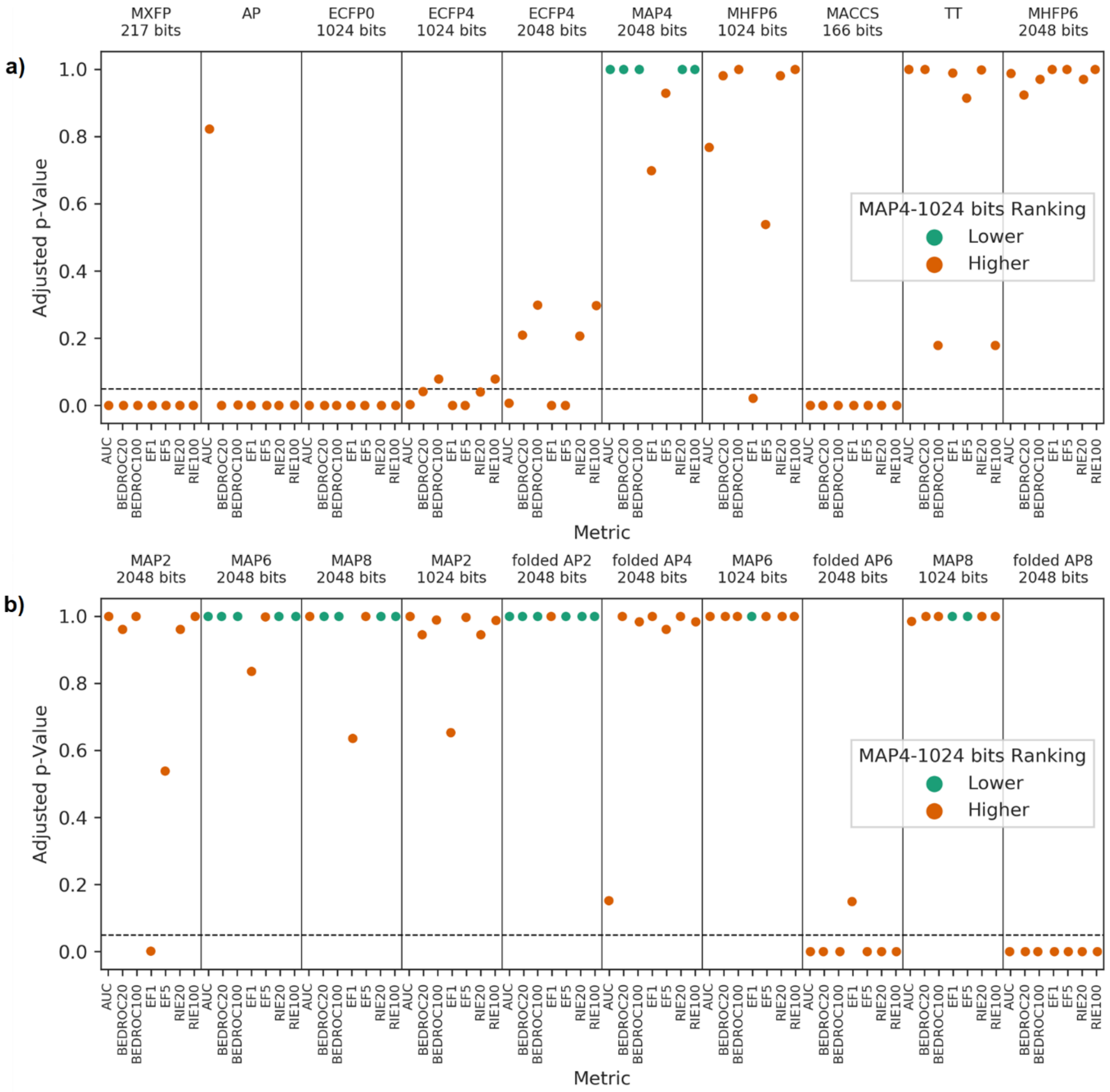

Figure S3. a) Relative ranking and p-values of and MAP4-1024 compared to MXFP-217, AP, ECFP0-1024, ECFP4-1024, ECFP4-2048, MAP4-2048, MHFP6-1024, MACCS-166, TT, and MHFP6 in the Riniker fingerprint benchmark when using only the DUD, MUV, and ChEMBL datasets. b) Relative ranking and p-values of and MAP4-1024 compared to MAP2-2048, MAP6-2048, MAP8-2048, MAP2-1024, foldedAP2-2048, foldedAP4-2048, MAP6-1024, foldedAP6-2048, MAP8-1024, and foldedAP6-2048 in the Riniker fingerprint benchmark when using only the DUD, MUV, and ChEMBL datasets. Orange color corresponds to MHFP6 being ranked higher than the other fingerprint, while green color indicates a lower ranking. P-values below 0.05 (dashed horizontal line) indicate significance.

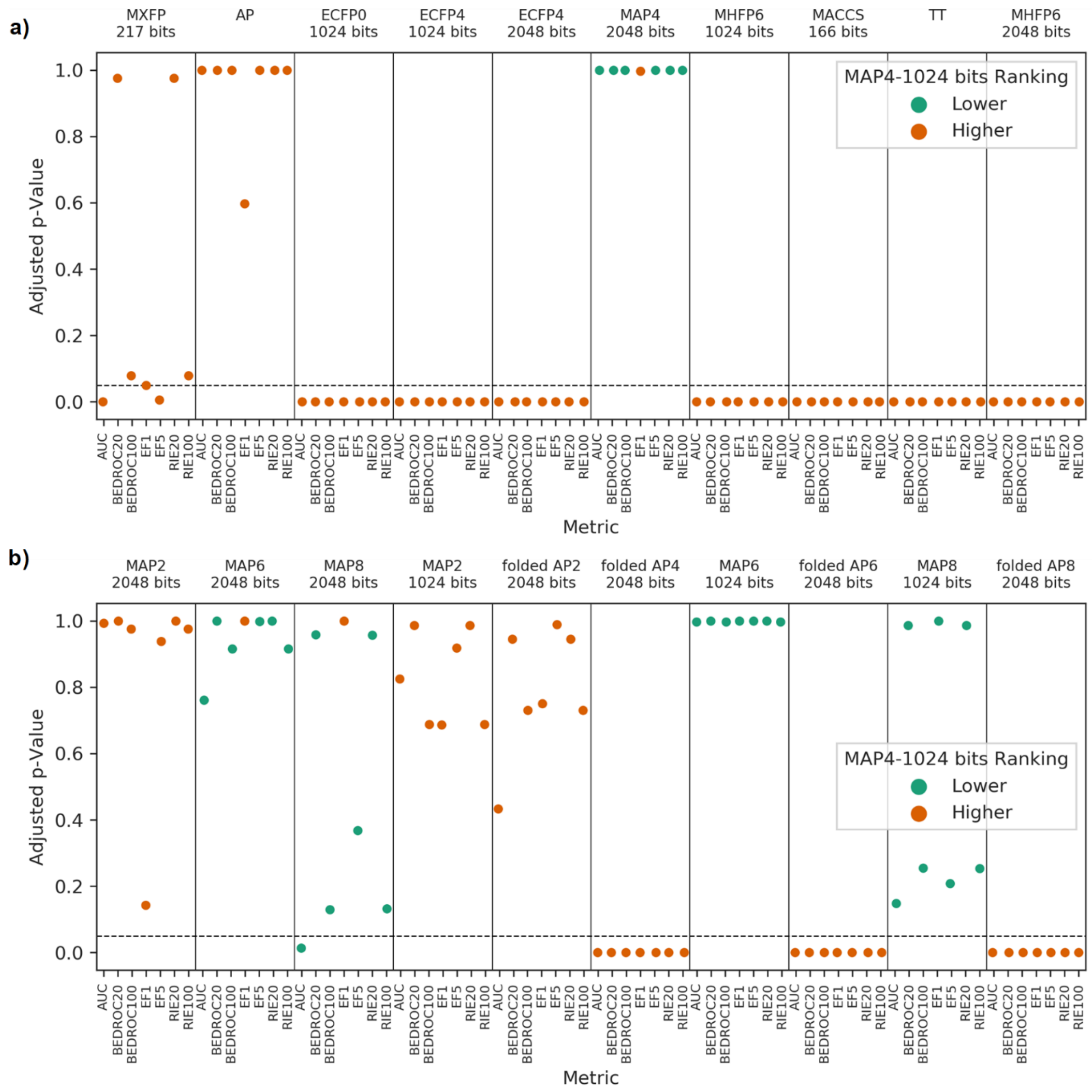

Figure S4. a) Relative ranking and p-values of MAP4-1024 compared to MXFP-217, AP, ECFP0-1024, ECFP4-1024, ECFP4-2048, MAP4-2048, MHFP6-1024, MACCS-166, TT, and MHFP6 in the Riniker fingerprint benchmark when using only the peptide datasets. b) Relative ranking and p-values of and MAP4-1024 compared to MAP2-2048, MAP6-2048, MAP8-2048, MAP2-1024, foldedAP2-2048, foldedAP4-2048, MAP6-1024, foldedAP6-2048, MAP8-1024, and foldedAP6-2048 in the Riniker fingerprint benchmark when using only the peptide datasets. Orange color corresponds to MHFP6 being ranked higher than the other fingerprint, while green color indicates a lower ranking. P-values below 0.05 (dashed horizontal line) indicate significance.

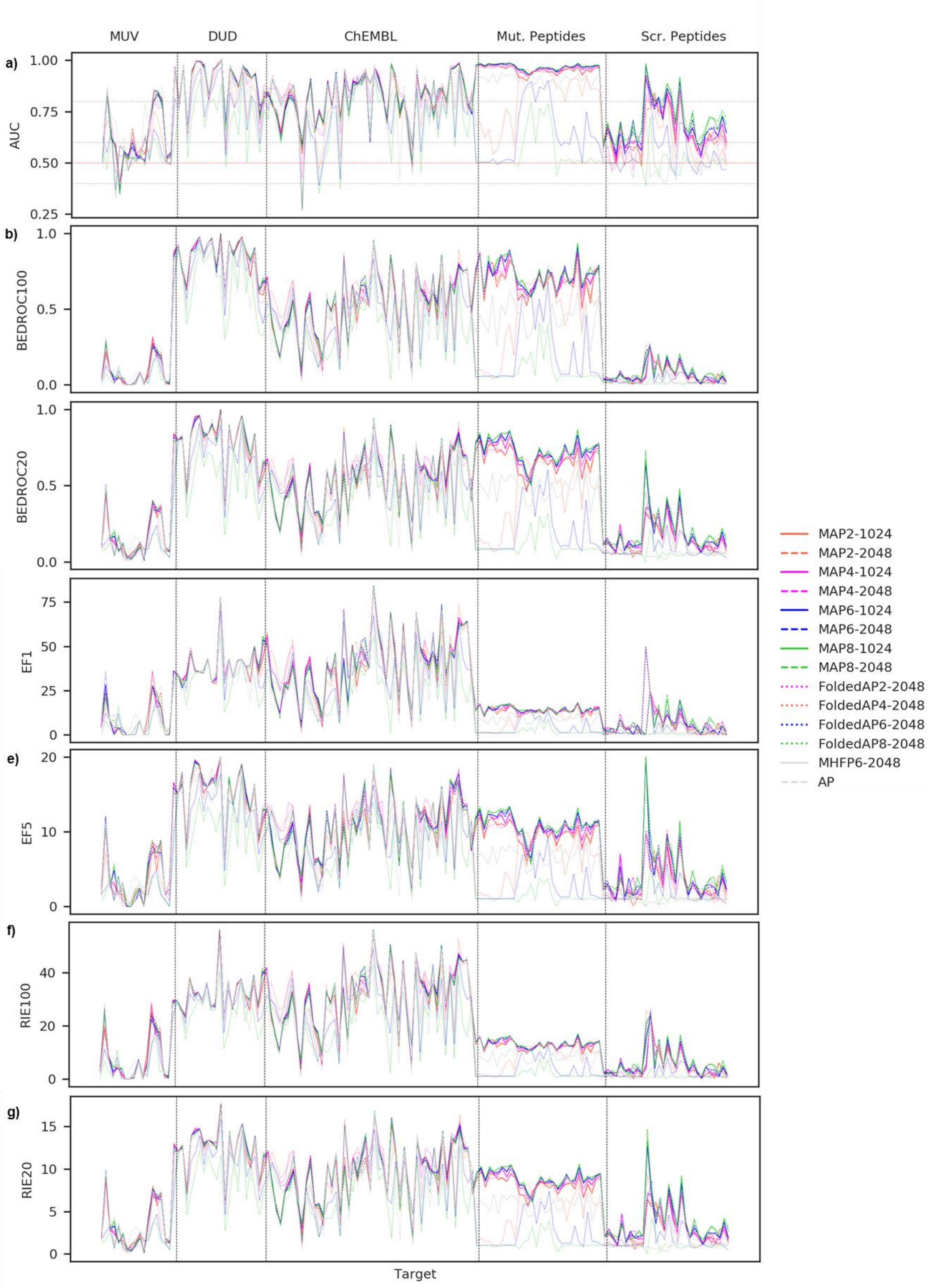

Figure S5. AUC (a), BEDROC100 (b) and 20 (c), EF1 (d) and 5 (e), RIE100 (f) and 20 (g) of AP2 (orange), AP4 (magenta), AP6 (blue), AP8 (green), in their 2014-dimensions (solid) and 2048-dimensions

(dashed) minhashed implementation (MAPs), and in their 2048-dimensions folded (dotted) implementation (foldedAPs). MHFP6 (solid) and AP (dashed) are reported in gray.

a) Example of structure found in the ECFP4 most populated bin (11333 molecules)

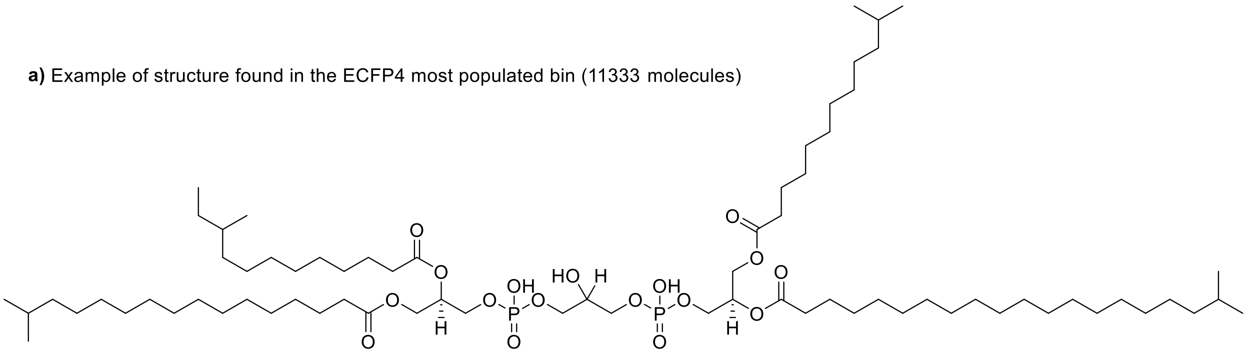

HMDB0072882

b) Example of structure found in the ECFP4 second most populated bin (5694 molecules)

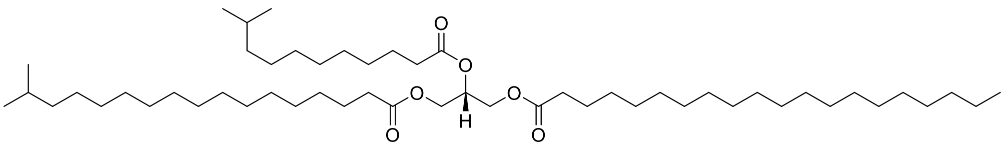

HMDB0108013

c) Example of structure found in the ECFP4 third most populated bin (3800 molecules)

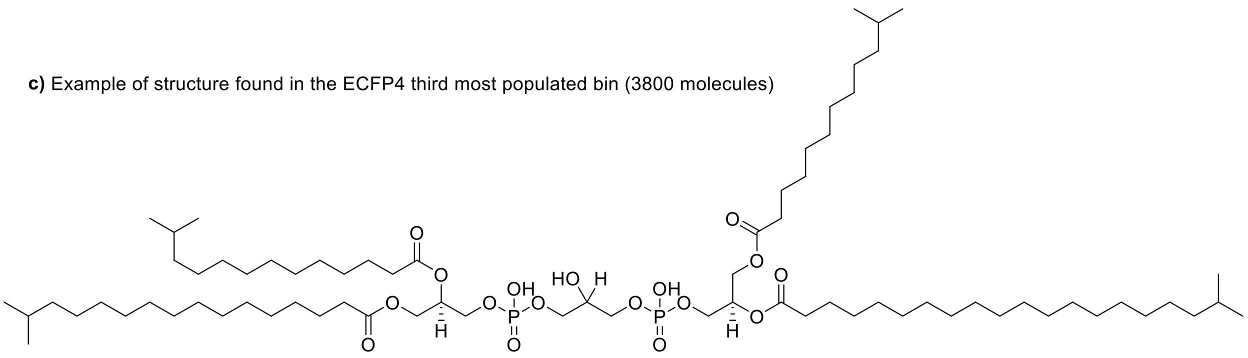

HMDB72885

Figure S6. Examples of structures found in the most (a), second-most (b) and third most (c) populated fingerprint value bins in the Metabolome ECFP4-1024 chemical space. The total amount of molecule present in the three fingerprint value bins is reported.

a) Example of structure found in the MHFP6 most populated bin (11,333 molecules)

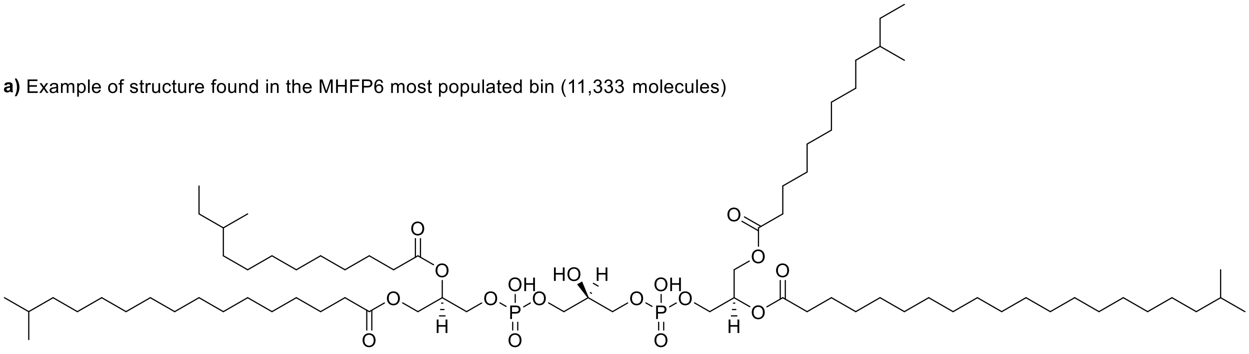

HMDB0072884

b) Example of structure found in the MHFP6 second most populated bin (5,694 molecules)

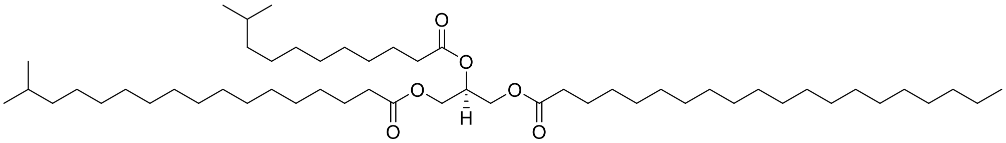

HMDB0062814

c) Example of structure found in the MHFP6 third most populated bin (3,800 molecules)

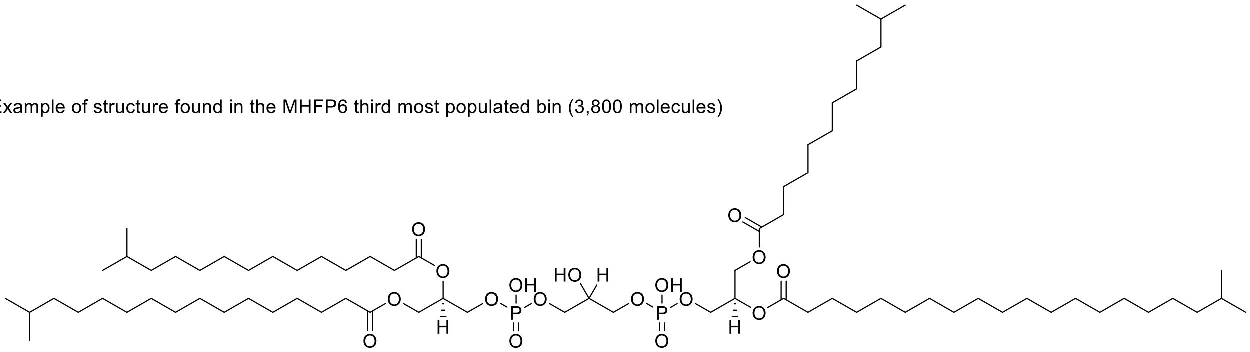

HMDB0072895

Figure S7. Examples of structures found in the most (a), second-most (b) and third most (c) populated fingerprint value bins in the Metabolome MHFP6-1024 chemical space. The total amount of molecule present in the three fingerprint value bins is reported.

a) Example of structure found in the TT most populated bin (381 molecules)

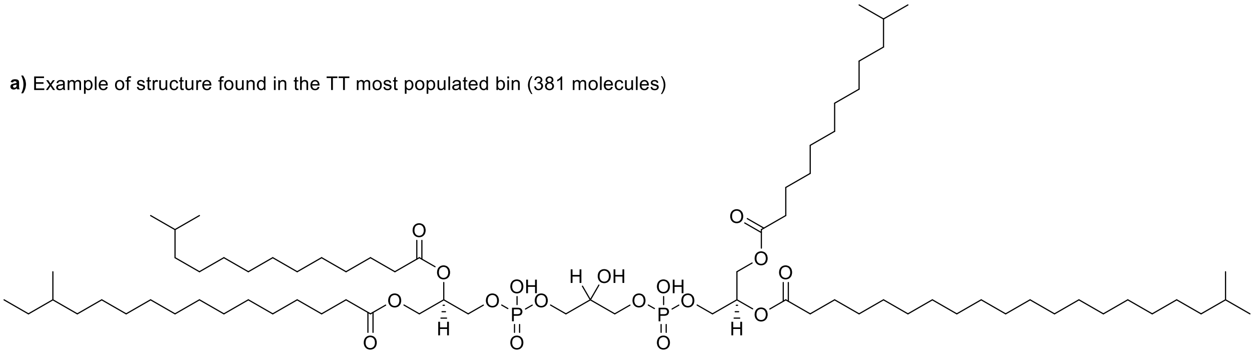

HMDB0072888

b) Example of structure found in the TT second most populated bin (378 molecules)

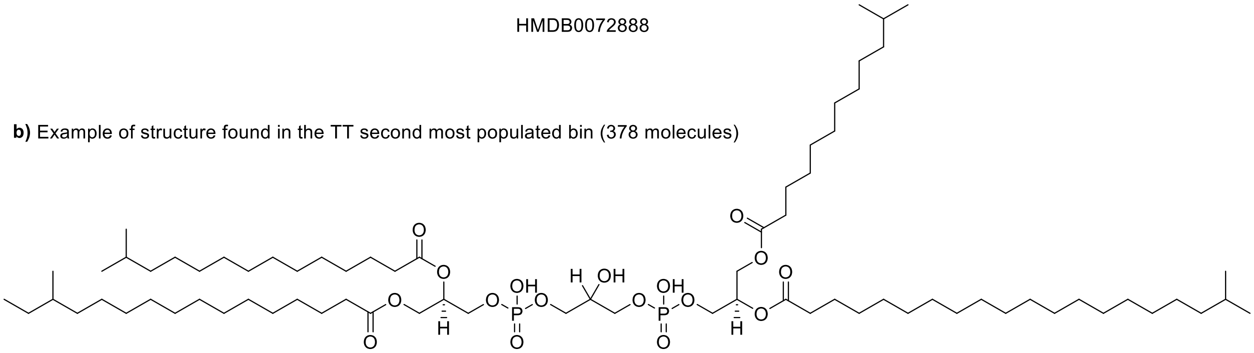

HMDB0072898

c) Example of structure found in the TT third most populated bin (374 molecules)

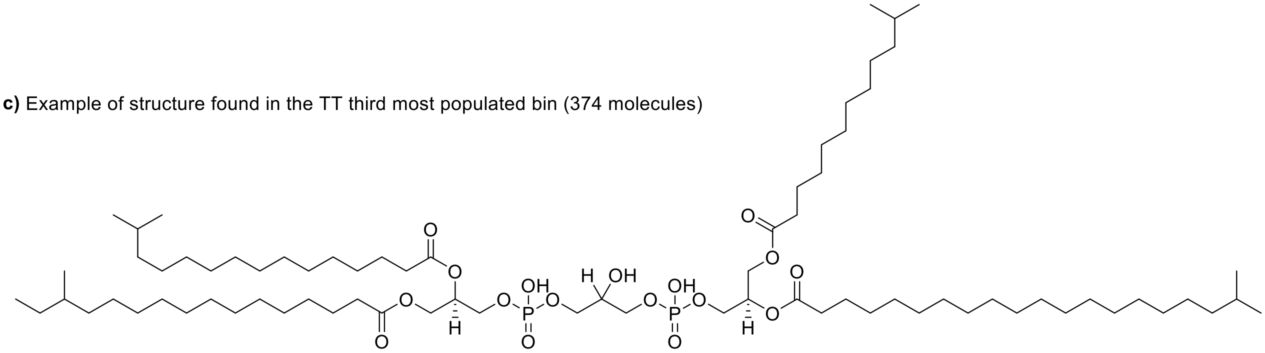

HMDB0072912

Figure S8. Examples of structures found in the most (a), second-most (b) and third most (c) populated fingerprint value bins in the Metabolome TT chemical space. The total amount of molecule present in the three fingerprint value bins is reported.
